# Mining Early Life Risk and Resiliency Factors and Their Influences in Human Populations from PubMed: A Machine Learning Approach to Discover DOHaD Evidence

**DOI:** 10.3390/jpm11111064

**Published:** 2021-10-22

**Authors:** Shrankhala Tewari, Pablo Toledo Margalef, Ayesha Kareem, Ayah Abdul-Hussein, Marina White, Ashley Wazana, Sandra T. Davidge, Claudio Delrieux, Kristin L. Connor

**Affiliations:** 1 Health Sciences, Carleton University, Ottawa, ON K1S 5B6, Canada; shrankhalatewari@cmail.carleton.ca (S.T.); ayeshakareem@cmail.carleton.ca (A.K.); ayahabdulhussein@cmail.carleton.ca (A.A.-H.); marina.white@carleton.ca (M.W.); 2 CONICET, National Science and Technology Council of Argentina, Buenos Aires C1425FQD, Argentina; pabloatm980@gmail.com (P.T.M.); cad@uns.edu.ar (C.D.); 3 Department of Psychiatry, McGill University, Montreal, QC H3A 0G4, Canada; ashley.wazana@mcgill.ca; 4 Women and Children’s Health Research Institute, University of Alberta, Edmonton, AB T6G 1C9, Canada; sdavidge@ualberta.ca; 5 DIEC—Electric and Computer Engineering Department, Universidad Nacional del Sur, Bahía Blanca B8000, Argentina

**Keywords:** Developmental Origins of Health and Disease, developmental programming, machine learning, natural language processing, text mining

## Abstract

The Developmental Origins of Health and Disease (DOHaD) framework aims to understand how early life exposures shape lifecycle health. To date, no comprehensive list of these exposures and their interactions has been developed, which limits our ability to predict trajectories of risk and resiliency in humans. To address this gap, we developed a model that uses text-mining, machine learning, and natural language processing approaches to automate search, data extraction, and content analysis from DOHaD-related research articles available in PubMed. Our first model captured 2469 articles, which were subsequently categorised into topics based on word frequencies within the titles and abstracts. A manual screening validated 848 of these as relevant, which were used to develop a revised model that finally captured 2098 articles that largely fell under the most prominently researched domains related to our specific DOHaD focus. The articles were clustered according to latent topic extraction, and 23 experts in the field independently labelled the perceived topics. Consensus analysis on this labelling yielded mostly from fair to substantial agreement, which demonstrates that automated models can be developed to successfully retrieve and classify research literature, as a first step to gather evidence related to DOHaD risk and resilience factors that influence later life human health.

## 1. Introduction

The Developmental Origins of Health and Disease (DOHaD) hypothesis posits that environmental exposures in early life programme health across the lifecycle [1]. To understand health determinants and the pathogenesis of diseases and disorders [2], it is necessary to not only consider early-life events, but also integrate knowledge on social, environmental, and biomedical health factors. Such an approach will enable better prediction of developmental trajectories and health risks, and identification of protective factors that promote resiliency and optimise health outcomes. Quantifying the complex interactions between early-life exposures and health and disease outcomes is a barrier to consolidating interdisciplinary knowledge in the DOHaD field, and, to-date, no comprehensive list of influential exposures and their interactions has been developed, limiting our ability to predict risk and resilience trajectories in humans [3].

The lack of efficient methods for gathering, analysing, and disseminating information from the multitude of multidisciplinary publications in the DOHaD field is a key barrier to synthesising knowledge on factors that promote risk and resiliency. Advances in techniques that allow for automated retrieval of relevant information and literature, including text mining, have been successfully used in the biomedical, life science, chemistry, and genetics fields [4,5,6]. In text mining, large bodies of text are navigated to identify patterns and extract relevant information, and machine learning (ML) and natural language processing (NLP) can further aid the process of mining text in scientific publications [7]. Papers identified as relevant can then be organised into groups of similar data items, using topic modelling or clustering, to make inferences and generate analysis at a broader semantic level [7,8].

Here, we aimed to incorporate text mining, ML, and NLP approaches to develop an automated process to capture information spanning the breadth of DOHaD literature, which constitutes a novel approach in this context. Our objective was to build a tool that can search, filter, and categorise information from primary literature in the DOHaD field, to enable the creation of an inclusive list of the early life exposures that most impact later life health outcomes. This research lays the foundation for a comprehensive DOHaD database that will integrate knowledge on the social, environmental, and biomedical determinants of health, and serves as the first step in the development of a tool that can mine data from the literature and integrate this with personalised information to predict developmental outcomes, health risks, and identify protective factors that ensure resiliency.

## 2. Methods

**Figure 1:**
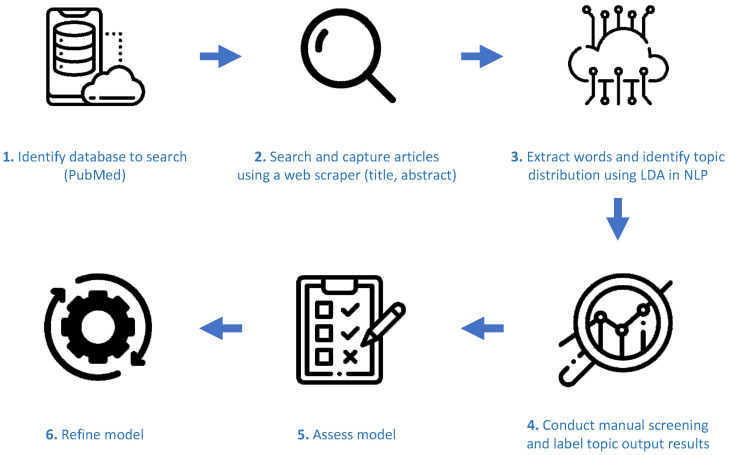
Overview of the text mining approach. Icons from www.flaticon.com/, accessed on 2 June 2021.

The creation and refinement of the text-mining model was composed of five processes (Figure 1). The methods and materials provided follow previously proposed guidelines for model development, evaluation, and reporting (Supplementary Table S1) [9,10]. The source code used to generate the models and visualisations can be found at the THRIVE repository (https://gitlab.com/papablo/thrive). Here, we provide instructions for how to create a containerised environment to run the data scraping and model creation and generate the intended visualisations.

### 2.1. Part 1: Text-Mining Model 1

To first create the foundation of a text-mining model, we narrowed our scope to the largest research database, PubMed. Although DOHaD research is multidisciplinary, as a proof-of-concept, we aimed to focus on a single research database, tailoring the tool to one search engine and its format. There were no date restrictions added to the model as the intention was to text-mine any article that fell into the search string parameter. The following search string, developed through preliminary research and expert consensus [3], was used to capture articles grounded in DOHaD: (maternal* OR paternal*) AND (weight OR obes* OR nutrition OR diet* OR stress OR social support) AND (child OR infant) AND (programming AND development); mother-child relation* AND programming; child* AND development AND programming AND (stress OR depression OR anxiety OR sensitivity OR temperament) AND mental health; parent-child relation* AND programming; programming AND *natal; development* AND origins AND programming; development* origins of health and disease; (maternal* OR paternal*) AND (gene* OR immune* OR metabol* OR inflam* OR brain OR neuro* OR cardio* respiratory) AND (development OR growth OR programming) AND (child OR infant).

We automatically captured all the articles from PubMed that met the criteria above, using a web scraper using Python and Selenium [11]. In order to build a latent Dirichlet allocation (LDA) model [10] as part of NLP [12], which uses the frequency of words to generate topics to describe a large collection of documents [10], three components were built into the model:

1. A corpus (or body of text, which in this case are the titles and abstracts of the articles);
2. A dictionary, associating each word to a unique numeric ID;
3. A number of topics to be extracted from our documents.

Using the mentioned corpus, the LDA process was used to extract words that frequently appear together, along with the frequencies of these words in relation to broader topics. The model output provided a statistical (parametric or otherwise) distribution of topics in the corpus. The number of topics was arbitrary and was set prior to building the model [8]. The LDA model also provided a measure of the level of similarity between a document (one that the model used for training or a new one acquired from scraping) and those in the corpus.

### 2.2. Part 2: Manual Labelling Text-Mining Model 1 Results

Labels for the topics generated by Model 1 were manually assigned based on the 30 most frequent and relevant terms which formed each topic. Labelling was done to identify domains/groups for each topic based on the frequency and types of terms captured within a particular topic. The terms within each topic were assessed to determine which field or domain within DOHaD the terms broadly fell into.

### 2.3. Part 3: Manual Assessment of Model 1 Accuracy–Classifying Model 1 Articles

**Figure 2:**
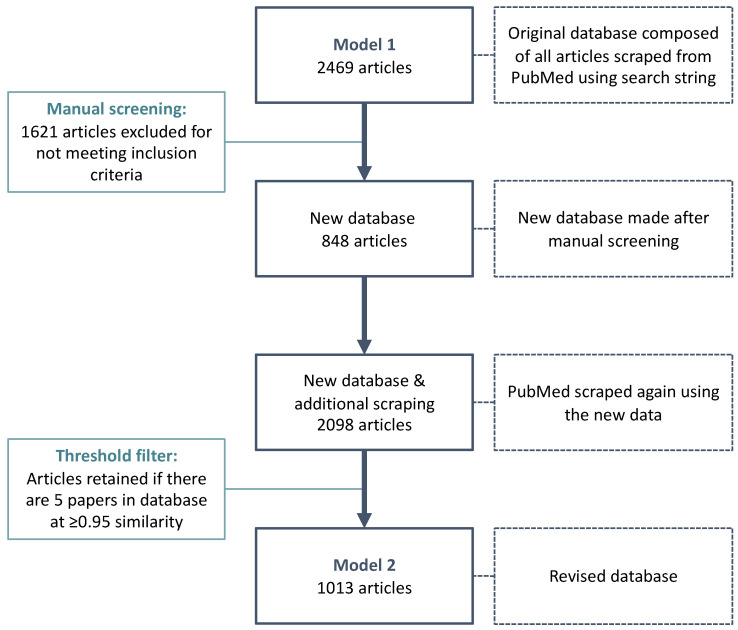
Text mining model development flowchart.

To refine our text-mining model to ensure it was capturing and assessing relevant primary human DOHaD articles, we performed a manual screening of the 2469 article results from Model 1 (Figure 2). The title and abstract for each of the 2469 articles were exported as a .csv file and screened. The exclusion criteria included non-primary articles (e.g., review articles and editorials), non-English language articles, and articles with missing/inaccessible abstract data (Table 1). Animal studies, whilst essential for understanding the mechanisms linking early life exposures to later life health outcomes and for developing pre-clinical interventions, were excluded from our model. We took this approach because of remaining limitations on our ability to predict which exposure-outcome relationships in animal models are predictive of human responses to the same exposures, and/or carry the same influence (weight) on health risk and resiliency in humans. [13,14,15,16]. A reliable method to evaluate the translatability of animal models to humans is necessary before animal studies can be included and effects weighted appropriately in a tool designed to predict human health outcomes [15]. Further, because the primary goal of this tool is not to interrogate the mechanisms linking early-life environments and later health, but to synthesise the breadth of knowledge on exposure-outcome associations in human populations, the inclusion of animal studies in our analysis would have unnecessarily widened the scope of the study as well as the amount of irrelevant latent topics that arise. After applying exclusion criteria to the manual screening of the 2469 articles scraped from Model 1, 848 articles were classified as “Related to our DOHaD focus” (Figure 2).

### 2.4. Part 4: Text-Mining Model 2

The first corpus for Model 2 was created using the titles and abstracts of the 848 articles that were manually classified as “Related to DOHaD” from our manual screening results of Model 1. To build a revised model based on articles “related to DOHaD”, PubMed was scraped again using the 848-article corpus. From this, a total of 2098 articles were obtained (Figure 2). Our final goal is to develop a model that would autonomously identify new articles related to DOHaD, in this and other repositories, and to automate the update process. For this reason, we further refined the model restricting the inclusion criteria (i.e., relevance to DOHaD). This was achieved through incremental training cycles that progressively refined and enlarged the underlying model. In each training cycle, a batch of 100 sample articles were processed, passing through the corpus, undergoing data transformation from raw format into readable format, fitting model parameters, and testing the trained learning scheme against the reference corpus. To do this, we added a similarity threshold filter (0.95) to assess the similarity of a new article to the 848 articles in our database. This meant that to be retained in the corpus and used for future training of the model, newly captured articles needed to be at least 95% similar in frequency of words and topics similar to at least five articles already present in the accepted corpus.

The similarity threshold of 0.95 was identified, through exploratory analysis, as the threshold at which relationships between candidate and already analysed and accepted papers were visible. The 100-sample article limit was chosen to balance the desire for a large number of articles in the training batches versus the capabilities of the computational resources available.

### 2.5. Part 5: PyLDAvis Visualization

A principal component analysis (PCA) and plot was produced with the standard pyLDAvis tool (v3.3.1) to visualise the results generated from the topic modelling and better understand the relationships between the words and the created clusters. The plot grouped the words extracted from articles into a predetermined number of topics based on frequency of the words in the captured articles. A topic number would be considered optimal if it produced an output with distinct topics (identified by the terms captured within), minimal overlapping of topics, and a fairly even distribution of keywords across the topics. To first explore the different topics and pick an optimal topic value into which words would be clustered, a PC plot was generated for topic group values 20, 25, 30, 60, 90, 120 and 200. These values were chosen arbitrarily, where results were manually evaluated and assessed based on general observations of keywords within topics, overlap, and overall distribution of keywords. The 200-topic value was chosen as an excessive number of topics to showcase the complexity of the model in which interpretation and subjective validation of topics is difficult [17] (see Supplementary Table S2 for 200-topic output).

The PC plots can be interpreted as follows: Similar words that appear together most frequently form a topic. The size of the circle represents the frequency of terms that the topic spans over the entire corpus. The distance between circles (as measured from their centre points) indicates the (dis)similarity between topics. That is, the closer two circles are to each other, the more similar the contained words within each topic. Circles that overlap or are separated by short distances described topics which were similar to each other. For each topic, a histogram is produced that displays the top 30 most relevant terms within that topic. The term frequency within that topic is represented by the horizontal red bars and the blue bars represent the frequency of the term across the whole corpus (i.e., overall term frequency).

### 2.6. Part 6: Consensus Evaluation of Model 2

Lastly, we sought to assess the quality of the topic labelling results from Model 2 against expert consensus. We asked 23 experts from different disciplines across the DOHaD field, recruited through the DOHaD Canada membership, to propose a unique label for each of the 30 topics identified by Model 2, based on their most relevant terms extracted by the latent information analysis. This group had expertise in development, maternal health/obstetrics, fetal/placental development and health, child health/paediatrics, psychology/psychiatry, neuroscience/neurodevelopment/neurological diseases, (epi)genetics/molecular medicine, anthropology, nutrition, metabolism/metabolic disease, public health/health policy, and social determinants of health. Where respondents used different terms to refer to the same latent topic, two authors (KLC and MW) reconciled semantic agreement between topics (for example, classifying “allergies” and “allergy”, or “endocrine” and “hormonal” as the same topic). We then calculated Cohen’s Kappa coefficient to assess consensus between experts on the appropriate labels for each of the 30 topics. This coefficient is widely used to measure inter-rater classification consensus. It is more robust than simple percent agreement since it considers agreements occurring by chance. Thus, the Kappa statistic requires a qualitative interpretation, which commonly regards values <0 as indicating no agreement, 0–0.20 as slight agreement, 0.21–0.40 as fair, 0.41–0.60 as moderate, 0.61–0.80 as substantial, and 0.81–1 as almost perfect agreement [18].

## 3. Results

### 3.1. Determining Topic Number from Text-Mining Model 1 to Best Capture DOHaD Domains

**Figure 3:**
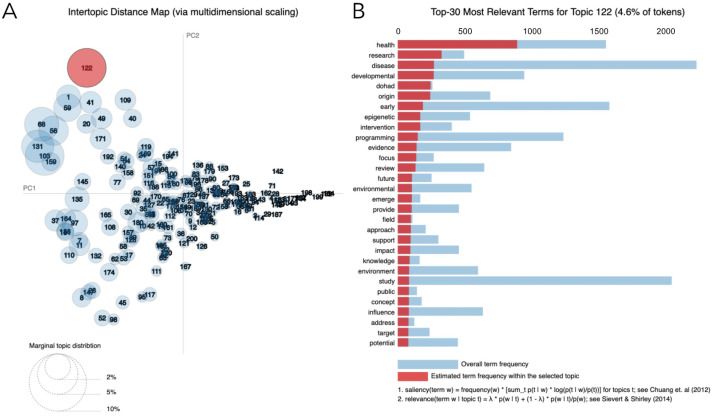
Results for text mining Model 1 for 200 topics via pyLDAvis. (**A**) PC plot and (**B**) histogram for 200-topic value from Model 1. (**A**) Each topic is represented as a circle (circle size = the frequency of terms that the topic spans over the entire corpus). The distance between circles (topics) indicates their (dis)similarity (closer = more similar). (**B**) The histogram displays the top 30 most relevant terms for the selected topic (in this example, the red circle in panel **A**). The term frequency within that topic is represented by the horizontal red bars. The blue bars represent the frequency of the term across the whole corpus. PC1, principal component 1. PC2, principal component 2.

**Figure 4:**
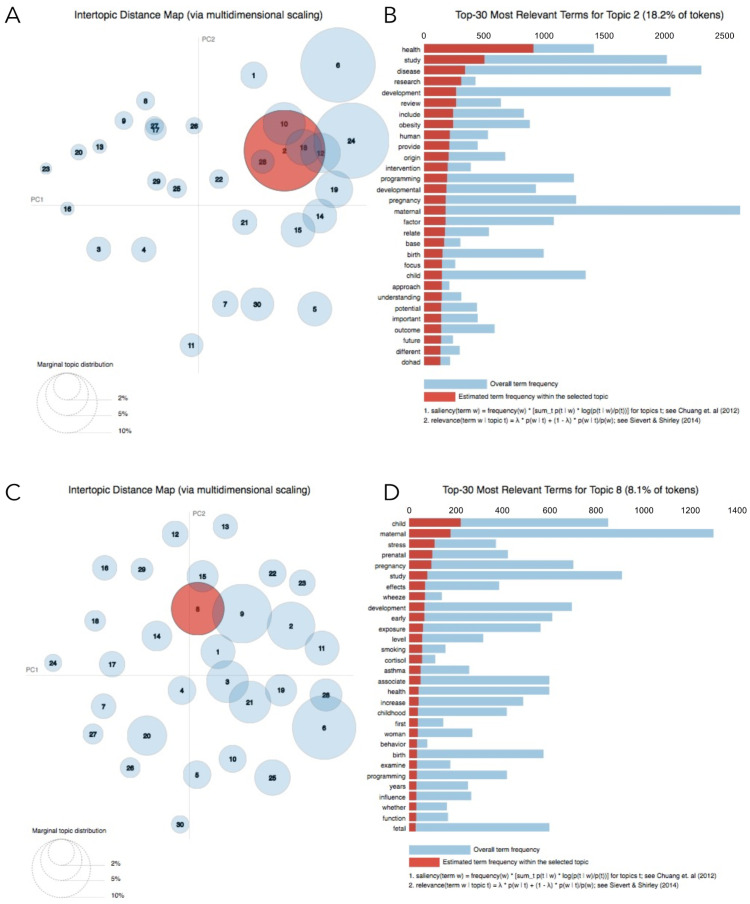
Results for text mining Model 1 (**A**,**B**) and Model 2 (**C**,**D**) for 30 topics via pyLDAvis. (**A**,**C**) Each topic in the PC plots are represented as a circle (circle size = the frequency of terms that the topic spans over the entire corpus). The distance between circles (topics) indicates their (dis)similarity (closer = more similar). (**B**,**C**) The histograms display the top 30 most relevant terms for the selected topic (in this example, the red circle in panels **A**,**C**). The term frequency within that topic is represented by the horizontal red bars. The blue bars represent the frequency of the term across the whole corpus. PC1, principal component 1. PC2, principal component 2.

The 2469 articles identified in Model 1 were extracted and scraped. Using LDA, topics were generated based on the distribution and frequency of terms in the titles and abstracts. High overlap between topics was observed for the 200-topic value (Figure 3), suggesting that subjective validation of each of the topics would be difficult. For the 120-, 90- and 60- topic values, there were several small topics with random terms and no evident DOHaD- related themes across topics, making it difficult to decipher what the topics represented (data not shown). For the 30-topic value, topics appeared better distributed based on subjective validation of the terms within each topic, and with minimal overlap, suggesting that the topics were more distinguishable from each other. For the 20- and 25-topic values, groups appeared ambiguous as the terms in many of the topics were related to general terminology (such as ‘offspring’, ‘fetal’, ‘maternal’, ‘disease’, and ‘developmental’) and not related to a specific field within DOHaD. Ultimately, the number of topics that best distinguishes the different domains within the captured DOHaD research was chosen to be 30. These 30-topic clusters contained broader and more inclusive groups of DOHaD related domains, while still being adequately specific and distinct from each other (Figure 4A).

### 3.2. Labelling Topics from Text-Mining Model 1

The results of the manual labelling, done by five experts in DOHaD with broad expertise, are listed in Table 2. Based on their assigned terms, topics were given labels such as ‘metabolic programming’, ‘drug-toxicant exposure’, ‘immunology’, ‘fetal development’, and ‘stress-related,’ amongst many others. Although some topics overlapped, these topics had different labels based on the frequency of terms making up the topics. This is because some the most frequent terms that were common between overlapping topics were ambiguous, and therefore were not used to create labels for these overlapping topics. For example, topics 18 and 12 overlap (Figure 4A) because the terms ‘exposure’ and ‘development’ are both amongst the most frequent terms in each of these topics. Nonetheless, topic 12 was labelled as ‘respiratory disorders’ and topic 18 was labelled as ‘immunology (infection, etc.)’ because the majority of the remaining relevant/frequent terms in these two topics were distinct, thus distinguishing the topics from each other. For topics where subjective validation was difficult because it was unclear what broad category the topic represented (e.g., topic 11 and 25), no label was created.

### 3.3. Determining Types and Relevance to DOHaD of Articles Scraped in Model 1

After manual abstract screening, 848 of the 2469 articles scraped in Model 1 were classified as “related to DOHaD” and met our inclusion criteria (Figure 2). The remaining 1621 (65.7%) articles scraped by Model 1 were excluded from the pool of articles used in subsequent steps, as they were: reviews, commentaries, protocols, or lectures (n = 824); animal studies (n = 392); not related to DOHaD (n = 255); non-primary articles (n = 72); non-English articles in a different language (n = 52); systematic reviews/meta-analyses (n = 44%); or had no abstract access (n = 76). There was some overlap between the article types such that, for example, a non-primary study article may also have been an animal study. Based on these results, we concluded that our initial model could be further improved by creating more specific model parameters.

### 3.4. Topic Modelling of Text-Mining Model 2

A total of 2098 articles were obtained after scraping PubMed again with the revised Model 2. The threshold similarity function was repeated for these 2098 articles, and a final revised model containing 1013 articles was obtained (Figure 2). The PC plot for Model 2 showed that the size of the circles did not differ much from one another, suggesting that topics had a somewhat even distribution of terms (Figure 4B). Additionally, the distances between the circles (topics) on the PC plot were not large, suggesting that the topics were similar to each other because the most frequent and relevant terms that made up these topics were more related to each other. Less overlap between circles is seen in Model 2, which indicates that topics may represent terms that can be grouped into distinct categories within the DOHaD field.

### 3.5. Consensus Evaluation of Model 2

**Figure 5:**
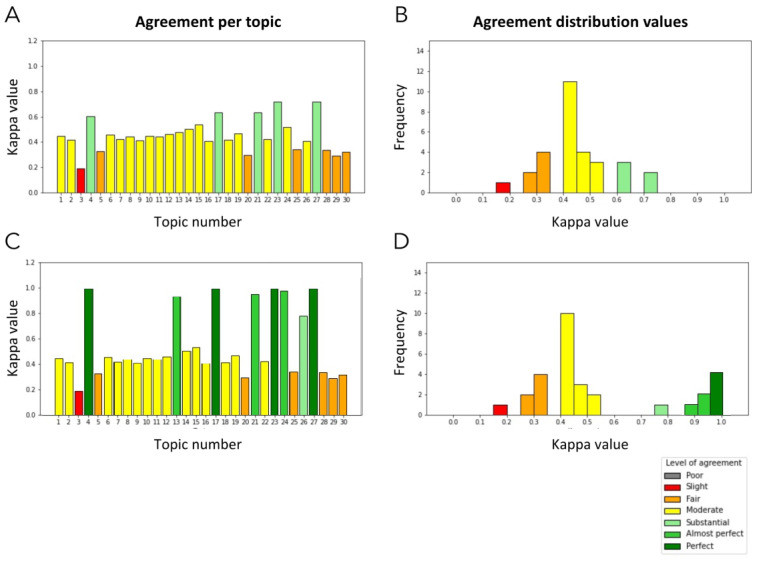
Consensus evaluation results comparing the topic labelling results from Model 2 against experts in the DOHaD field. Analysis of Cohen’s Kappa agreement statistics per topic (**A**) and by distribution (**B**) reveal that consensus among experts was substantial (n = 5), moderate (n = 18), fair (n = 6), and slight (n = 1) for the 30 topics. In eight topics (4, 13, 17, 21, 23, 24, 26 and 27), bimodal labelling was also considered (**C**), yielding better consensus measures (**D**).

In 22 cases, there was only one clear mode, while eight cases were labelled with two different topics (for instance topic 4 was interpreted either as “growth” or as “metabolic”). Whether these two labels indeed refer to different underlying topics or to different underlying aspects of the same topic may be the subject of debate. It is likely that this was attributable not to the NLP analysis but to the nature of the actual papers, which may span two topics within the DOHaD field. Thus, for these cases we performed two different analyses: one “stringent,” in which the consensus was evaluated regarding how many labels were coincident with only the most labelled of the modes (Figure 5A); and a more “lenient” analysis in which we regarded how many labels pertained to any of the two modes (Figure 5C). The distribution of topics across consensus measures are shown in Figure 5B,D.

## 4. Discussion

Here, we report on the development of a novel tool that uses text mining, ML, and NLP procedures to gather papers on topics relevant to a wide variety of DOHaD-related research articles. This is the first step towards mining data from primary research to aid in the development of a prediction tool that can identify early life health risk and resiliency factors. This proof-of-concept approach demonstrated that our trained model can scrape a large research database (PubMed) for primary human studies related to DOHaD, and identify and cluster key terms, based on their frequency, from the article titles and abstracts to create groups of topics.

Our first model (Model 1) was based on text mining only and had several drawbacks. Among them, studies unrelated to the broad DOHaD topic had to be manually screened out. Analysis of the clustering of latent topics in PCA space (in the top and bottom right-hand quadrants of Figure 4A) and the high overlap of topics indicate an uneven distribution of words across the topics constituting the corpus. This uneven distribution also suggests that Model 1 captured articles that were not related to DOHaD research, as illustrated by the small circles arising in the bottom left quadrant in Figure 4A. These circles represent topics that contain low-frequency words which differ vastly from most of the terms in the rest of the corpus.

In comparison, the more refined Model 2 narrowed down the scope of the search to focus on primary articles from human studies related to DOHaD, which were clearly and distinctly represented. This is rendered in the more evenly distributed circles in PCA space. Moreover, there was less overlap among the circles, and the circles were fairly centred, suggesting that terms were grouped into distinguishable topics. Lastly, there was less distance between circles, especially those at the far ends of the plot, indicating that these topics were more similar to each other, and the scope of the Model 2 search was narrowed down to more relevant studies (i.e., primary articles related to DOHaD).

After training the second model to have stricter similarity inclusion parameters, articles with a high degree of similarity in frequency of words and key terms to the training corpus could be retrieved. For example, as seen by the closer proximity of the topics, many of the prominent topics obtained were related to metabolic and physiologic health, a commonly studied exposure domain [3]. These findings indicate that the developed model could be tailored to focus on a specific field of research based on the types of articles that are fed into it, and in turn, extract meaningful data from that field.

Across clinical and academic settings, text mining strategies are emerging as a means to meet the growing need to remain up to date on the increasingly vast amount of research published across many scientific and clinical fields [17,19]. To date, there has only been one other study exploring machine learning techniques in the prenatal environmental exposures context, which mainly explored extracting and grouping studies based on methodology type [20]. Our study thus contributes another aspect to this field by introducing a proof-of-concept model for collating and discriminating studies based on specific topics within the DOHaD landscape. The steps of our proof-of-concept model follow a similar process used in studies that have also used LDA analysis and pyLDAvis for text-mining topic-distribution analysis in various fields [21,22,23,24]. Our findings are in line with the goals of other studies that aim to use LDA and topic-distribution analysis with different clustering visualisation methods [25,26,27]. These studies have emphasised the importance of having data visualisation models that interpret and synthesise extracted data from research articles for creation of quick-reference tools for clinicians or policy analysts. From a health policy perspective, developing unbiased and data-driven models for health prediction and promotion, such as the one we are proposing, can aid in decision-making processes while being accessible to a wide array of users. These models can consequently, provide quick synthesis of large datasets, and are cost-efficient [28].

There are important caveats to the application of our model that should be highlighted. Firstly, our revised model (Model 2) remains preliminary, and serves as a proof-of-concept that can function as a scraping tool to gather relevant and focused information. As multiple topics in the revised model were related to specific domains within DOHaD (such as the metabolic and physiologic health domain), our model requires further refinement of its parameters to filter and optimise the search strategy to ensure it is capturing a relevant and balanced representation of articles across all research domains in the field (including the nutrition, genetics, neurocognitive, and psychological health domains). Secondly, due to coding limitations, our model was designed to only scrape information from one database (PubMed) and, thus, applying this model to other databases (including those that have greater focus on research studies in sociological and psychological fields) is a necessary next step to ensure retrieval of relevant DOHaD articles across the breadth of health research fields. Lastly, extending the model to allow full text extraction, beyond the title and abstract scraping we have performed here, would allow us to more comprehensively capture methodology, results and statistical data [29], a requirement for identifying important variables related to health risk/resilience trajectories. It is worth mentioning that our methodology is fully adaptable to other domains, requiring only to determine an initial seed corpus (Model 1) and a manual screening out of undesired entries in this corpus, after which the same Model 2 automated building procedure can be performed.

Whilst our method can also be used to capture model organism literature related to DOHaD, the purpose of this study was to synthesise the breadth of DOHaD research in human populations. We envision our approach can facilitate researchers who aim to comprehensively capture and better understand the mechanisms underlying developmental programming, for the development of pre-clinical interventions based on data-driven models, and to better understand existing pre-clinical interventions aimed to prevent adverse outcomes or to optimise development and health trajectories. Of course, in doing so, one must be mindful of the variations in species, strain, study design and reporting, laboratory techniques, and timing/dosage of exposures and interventions in animal models, as these will influence the translatability of the findings to human populations [13,14,30]. This is of particular importance when considering the influence of early environments and exposures on later health, given the differences in pregnancy and developmental milestones between species that may determine the resulting phenotype. Although a ML tool like the one we present here can provide up-to-date topic modelling on both human and animal model studies, the results are complex and difficult to comprehend. Therefore, our strategy is to first focus on human studies, thoroughly understand the meaning of the results, and then in future research, widen the scope of the corpus to be analysed so that it includes animal models.

Moving forward, using keywords specific to different DOHaD domains and developed through field-expert consensus, multiple ML models can be developed to specifically target diverse research areas within DOHaD, building upon the same approach used here. This would ensure the capture of articles from research domains that are underrepresented in the DOHaD field, as we have previously identified [3]. It would also facilitate the construction of a database of primary DOHaD studies and enable easier assessment and synthesis of important data on risk and resiliency factors in the large body of DOHaD literature. The mined data could then be included in a repository for the development of a prediction tool based on early life environments. Such an approach will help to fill the unmet need of integrating risk and resiliency factors across social, environmental, and biomedical perspectives, to better understand early life exposures and subsequent health-outcome relationships. Further, the creation of a predictive tool will help inform the development of clinical and public health interventions in early life that can prevent adverse health outcomes and optimise health trajectories.

## Figures and Tables

**Table 1:** Manual Labelling Results for Model 1 of the Text-Mining Model.

Manual Labelling Results: Scraping Tool Articles	Number of Articles
Articles Scraped from Code That Were Found in PubMed Search of RER * Articles (Out of a Sample of 100)	75
Animal Studies	392
Reviews/Lectures/Commentaries/Protocols	824
Systematic Reviews and Meta-Analyses	44
Not a Primary Study Article	72
No Access/Information	76
Different Language	52
Not Related to DOHaD	255
Included Articles (Related to DOHaD According to Abstract Judgement)	848

* RER, rapid evidence review.

**Table 2:** Suggested labels for results for Model 1 of the text-mining model for 30 topics via pyLDAvis.

Topic Number	Suggested Label
1	Brain/Neuro Dysfunction
2	Health Research
3	Parent-Child Relationship
4	Global/Rural Socioeconomic Status
5	Auto-Immune Disorders (Allergies, Asthma, Wheeze, Eczema, etc.)
6	Metabolic Programming (Cardiovascular, Hypertension, Programming, etc.)
7	Blood/Hormone Levels (Serum Properties)
8	Infection/Inflammation (Cellular Level)
9	Pathogens
10	Hepatogenic secretions
11	None
12	Respiratory Disorders
13	Fatty Acids/Diet
14	Metabolic/Supplementation
15	Diabetes
16	Hospitalization
17	Development (Mutation, Underdeveloped)
18	Immunology (Infection, etc.)
19	Time Periods (Prenatal, Neonatal, Birth) (Possibly as n Exposure)
20	Steroids
21	Fetal Development
22	Drug/Toxicant Exposure
23	Cardiovascular Dysfunction
24	Stress-Related
25	None
26	Microbiome-related
27	Alcohol
28	Toxicants
29	Environmental
30	Childhood Disorders

## Data Availability

Data and code used in these processes are available at https://gitlab.com/papablo/thrive and further described in Supplementary Table S1.

## Supplementary Materials

The following are available online at https://www.mdpi.com/article/10.3390/jpm11111064/s1, Table S1: Model development and evaluation reporting, Table S2: 200-topic Model 2 output.
