# Supplementary material for: Mining Early Life Risk and Resiliency Factors and Their Influences in Human Populations from PubMed: A Machine Learning Approach to Discover DOHaD Evidence
Source: J Pers Med. 2021 Oct 22;11(11):1064. doi: 10.3390/jpm11111064 (PMC8621659; doi:10.3390/jpm11111064)
Supplement: Supplementary file 1 [file jpm-11-01064-s001.zip › Supplementary Table S1.pdf]

Table S1. Model development and evaluation reporting.

Checklist item	Author response
**1. Data sources**	
1a. Are all data sources listed and publicly available?	Yes, they are available at https://gitlab.com/papablo/thrive (model generation steps) and https://gitlab.com/papablo/thrive-consensus (consensus analysis).
1b. If using an external database, is an access date or version number provided?	PubMed was accessed on September 27, 2019 (initial article scraping) and February 2, 2020 (second scraping for incremental model).
1c. Are any potential biases in the source dataset reported and/or mitigated?	No
**2. Data cleaning**	
2a. Are the data cleaning steps clearly and fully described, either in text or as a code pipeline?	Yes
2b. Is an evaluation of the amount of removed source data presented?	No
2c. Are instances of combining data from multiple sources clearly identified, and potential issues mitigated?	All data came from a single source (PubMed).
**3. Data representation**	
3a. Are methods for representing data as features or descriptors clearly articulated, ideally with software implementations?	Each extracted document is processed so every term is taken to a root form, allowing the construction of a more precise model.
3b. Are comparisons against standard feature sets provided?	No
**4. Model choice**	
4a. Is a software implementation of the model provided such that it can be trained and tested with new data?	The method used to create the model can be adapted to be trained and tested on new data or for different research interests.
4b. Are baseline comparisons to simple/trivial models (for example, 1-nearest neighbour, random forest, most frequent class) provided?	No

4c. Are baseline comparisons to current state-of-the-art provided?	No
5. Model training and validation	
5a. Does the model clearly split data into different sets for training (model selection), validation (hyperparameter optimization), and testing (final evaluation)?	The parameters used in the creation of the models required some trial and error, but a satisfactory set of configurations was obtained. Given the nature of the models, the validation is based on supervision from experts.
5b. Is the method of data split (data splitting for example, random, cluster- or time-based splitting, forward cross-validation) clearly stated? Does it mimic anticipated real-world application?	This kind of model construction does not require data splitting (train-test-validation).
5c. Does the data splitting procedure avoid data leakage (for example, is the same composition present in the training and test sets)?	Not applicable
6. Code and reproducibility	
6a. Is the code or workflow available in a public repository?	Yes, the model generation steps are at https://gitlab.com/papablo/thrive and the consensus analysis is at https://gitlab.com/papablo/thrive-consensus
6b. Are scripts to reproduce the findings in the paper provided?	The order of execution is indicated with the name of the files.
